# Weighted Gene Coexpression Network Analysis to Construct Competitive Endogenous RNA Network in Chromogenic Renal Cell Carcinoma

**DOI:** 10.1155/2021/5589101

**Published:** 2021-06-10

**Authors:** Yong-Bo Chen, Liang Gao, Jin-Dong Zhang, Jiang Guo, Ping-Hong You, Liang-You Tang, Ying-Wen Liu

**Affiliations:** ^1^Department of Urology, People's Hospital of Deyang City, 173# Northern Taishan Road, Deyang 618000, China; ^2^Department of Urology, The Second Affiliated Hospital of Chongqing Medical University, 74# Linjiang Road, Chongqing 400010, China; ^3^Department of Urology, The First Affiliated Hospital of Chongqing Medical University, 1# Youyi Road, Chongqing 400042, China; ^4^Department of Laboratory Medicine, People's Hospital of Deyang City, 173# Northern Taishan Road, Deyang 618000, China

## Abstract

**Aim:**

This study is aimed at constructing the competing endogenous RNA (ceRNA) network in chromophobe renal cell carcinoma (ChRCC).

**Methods:**

Clinical and RNA sequence profiles of patients with ChRCC, including messenger RNAs (mRNAs), microRNAs (miRNAs), and long noncoding RNAs (lncRNAs), were obtained from The Cancer Genome Atlas (TCGA) database. “edgeR” and “clusterProfiler” packages were utilized to obtain the expression matrices of differential RNAs (DERNAs) and to conduct gene ontology (GO) and Kyoto Encyclopedia of Genes and Genomes (KEGG) analyses. Weighted gene coexpression network analysis (WGCNA) was performed to screen the highly related RNAs, and miRcode, StarBase, miRTarBase, miRDB, and TargetScan datasets were used to predict the connections between them. Univariate and multivariate Cox proportional hazards regressions were performed in turn to elucidate prognosis-related mRNAs in order to construct the ceRNA regulatory network.

**Results:**

A total of 1628 DElncRNAs, 104 DEmiRNAs, and 2619 DEmRNAs were identified. WGCNA showed significant correlation in 1534 DElncRNAs, 98 DEmiRNAs, and 2543 DEmRNAs, which were related to ChRCC. Fourteen DEmiRNAs, 113 DElncRNAs, and 43 DEmRNAs were screened. Nine mRNAs (ALPL, ARHGAP29, CADM2, KIT, KLRD1, MYBL1, PSD3, SFRP1, and SLC7A11) significantly contributed to the overall survival (OS) of patients with ChRCC (*P* < 0.05). Furthermore, two mRNAs (CADM2 and SFRP1) appeared to be independent risk factors for ChRCC.

**Conclusion:**

The findings revealed the molecular mechanism of ChRCC and potential therapeutic targets for the disease.

## 1. Introduction

As one of the three major renal cell carcinoma histological subtypes, chromophobe renal cell carcinoma (ChRCC) accounts for 4%–5% of renal cancer cases [1]. The average diagnostic age of ChRCC is 58 years, and most patients are male [2]. Most patients with ChRCC have good prognoses with 5-year survival rates of 78%–100%. However, metastases still occur in about 6%–7% of patients and usually affect the liver or lungs [3]. Furman et al. and Panel et al.'s tumor classification schemes have already been proposed for use in staging ChRCC over the past decades [4, 5]. However, considering the ambiguity of the grading criteria and the lack of applicability to the characteristics of the nucleus of ChRCC, their prognostic value appears to have been overestimated [6, 7]. In order to better standardize treatment and improve patient prognosis, it is critical to elucidate highly specific biomarkers and effective therapeutic targets. In 2011, Salmena et al. described the competing endogenous RNA (ceRNA) hypothesis, which reexplored the regulatory function of long noncoding RNAs and the potential network between messenger RNAs (mRNAs), microRNAs (miRNAs), and long noncoding RNAs (lncRNAs) [8]. As a key element in the ceRNA network, miRNAs could simultaneously be competitively antagonized by lncRNA, mRNA, and other RNAs through shared microRNA response elements (MREs). Overexpressed MRE-containing transcripts (so-called “RNA sponges”) could affect expression by absorbing multiple miRNAs connected to mRNAs [9–11]. This molecular internal regulation mechanism plays an important role in the occurrence and development of multiple cancers [12]. The Cancer Genome Atlas (TCGA) database, established by the National Cancer Institute and the National Human Genome Research Institute, has collected numerous genomic, epigenomic, transcriptomic, and proteomic data for 33 cancer types [13, 14], facilitating exploration of the ceRNA network in ChRCC and the identification of prognostic-related biomarkers.

## 2. Methods

All clinical and RNA sequence profile data of patients enrolled in TCGA database before May 2020, including mRNA, miRNA, and lncRNA matrices, were completely downloaded and extracted from the dataset (https://portal.gdc.cancer.gov/). Inclusion criteria stipulated that the clinical data of every sample should, at least, include the patient's survival status and survival time. The R version 3.6.0 software was used for all statistical analyses. As a public database was used, additional approval from an ethics committee was not required.

The “edgeR” package of R (version 3.6.0) was used to elucidate and compare the DElncRNAs, DEmiRNAs and DEmRNAs of normal and cancer samples. Log2FC > 2 and FDR < 0.05 were considered statistically significant. We preformed gene ontology (GO) and Kyoto Encyclopedia of Genes and Genomes (KEGG) analyses using the “clusterProfiler” package (with *P* < 0.05 as significant) to construct the pathway-gene and pathway-pathway networks [15].

After verifying and confirming the optimal soft threshold, we conducted weighted gene coexpression network analysis (WGCNA) using the “WGCNA” package. RNAs were classified into different color modules according to the connectivity and synergy between them. In selecting the RNAs for next analyses, connectivity was established between each module and the relevant ChRCC trait.

The lncRNA-miRNA matrices in selected modules were predicted and simplified in miRcode (http://www.mircode.org/) and their associations obtained. These miRNAs were predicted using StarBase (http://starbase.sysu.edu.cn/), miRTarBase (http://mirtarbase.mbc.nctu.edu.tw/), miRDB (http://www.mirdb.org/), and TargetScan (http://www.targetscan.org/) datasets in order to obtain their target mRNAs. The mRNAs from selected modules were combined with the target mRNAs to exclude unrelated mRNAs.

**Figure 1:**
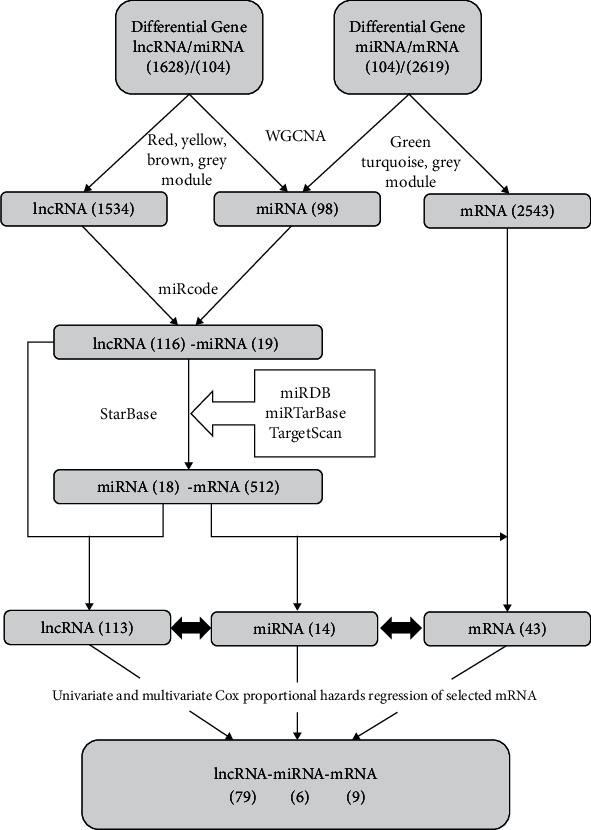
The flow chart of this study.

Finally, univariate and multivariate Cox proportional hazards regressions were performed in turn using the “survival” package of R to elucidate the most significant independent risk factor mRNAs associated with the OS of patients with ChRCC. Sample scores were compared to the median risk score and divided into high-risk and low-risk groups. ROC and C-indices were used to evaluate the stability and reliability of the mRNA-based prognostic model. The detailed flow chart is presented in Figure 1.

Based on the elucidated relationships between lncRNAs-miRNAs and miRNAs-mRNAs and the Cox results, we were able to derive the lncRNAs-miRNAs-mRNAs competing endogenous network. The Cytoscape software (version of 3.6.1) was used to visualize the ceRNA network. The Kaplan–Meier curves were used to analyze the reliability with which each RNA in the ceRNA network was able to predict the patient's OS (with *P* < 0.05 indicating significant reliability).

## 3. Results

The lncRNA, miRNA, and mRNA expression matrices of the 89 patients (24 normal and 65 with ChRCC) were downloaded from TCGA dataset. Patients' clinicopathological characteristics are presented in Table 1. The univariate and multivariate Cox proportional hazards regressions of patients' clinical data revealed that none of those characteristics were significant independent risk factors associated with their OS (Table 2).

**Figure 2:**
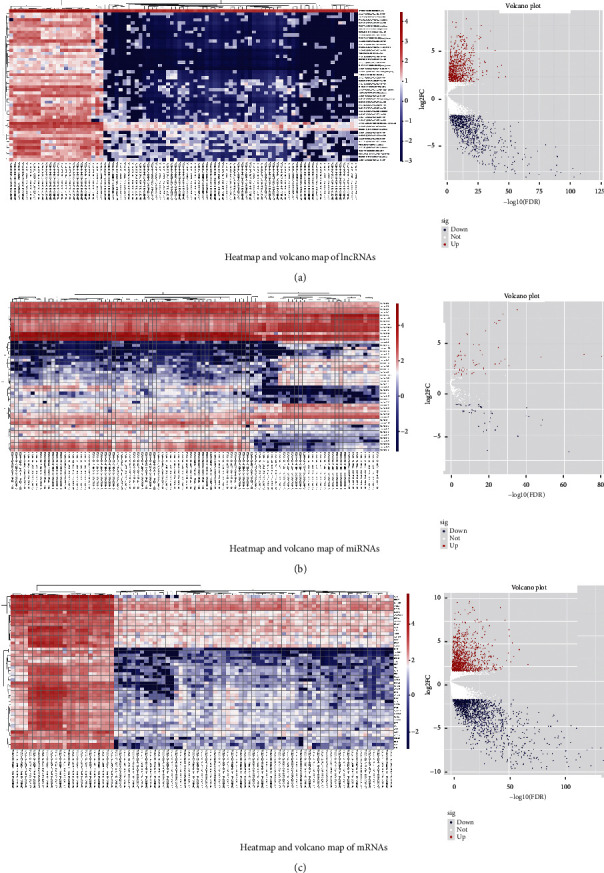
Heatmap and volcano map of (a) lncRNAs, (b) miRNAs, and (c) mRNAs.

**Figure 3:**
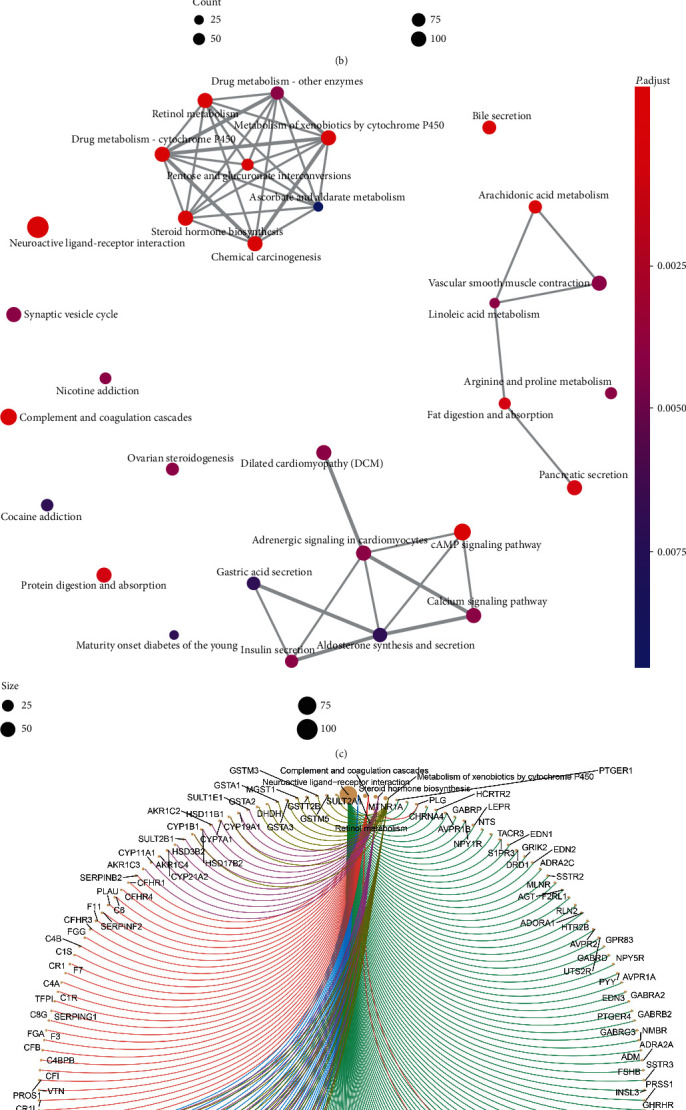
(a) GO and (b) KEGG analyses, (c) pathway-pathway network, and (d) pathway-gene network based on the 2619 DEmRNAs.

Firstly, 1628 DElncRNAs (763/865, up/down), 104 DEmiRNAs (61/43, up/down), and 2619 DEmRNAs (1103/1516, up-/down-DEmRNAs) were elucidated. Their volcano maps and heatmaps are presented in Figures 2(a)–2(c). GO analysis showed that the top five functions of the 2619 DEmRNAs focused on organic anion transport, regulation of membrane potential, regulation of ion transmembrane transport, modulation of chemical synaptic transmission, and regulation of transsynaptic signaling (Figure 3(a)). Meanwhile, the top five KEGG pathways of these DEmRNAs were enriched in neuroactive ligand−receptor interaction, cAMP signaling pathway, complement and coagulation cascades, retinol metabolism, and chemical carcinogenesis (Figure 3(b)). Insulin secretion and connection between pathways were presented in the pathway-pathway network (Figure 3(c)). In the pathway-gene network, multiple RNAs were related to five pathways: complement and coagulation cascades, metabolism of xenobiotics by cytochrome P450, neuroactive ligand−receptor interaction, retinol metabolism, and steroid hormone biosynthesis (Figure 3(d)).

**Figure 4:**
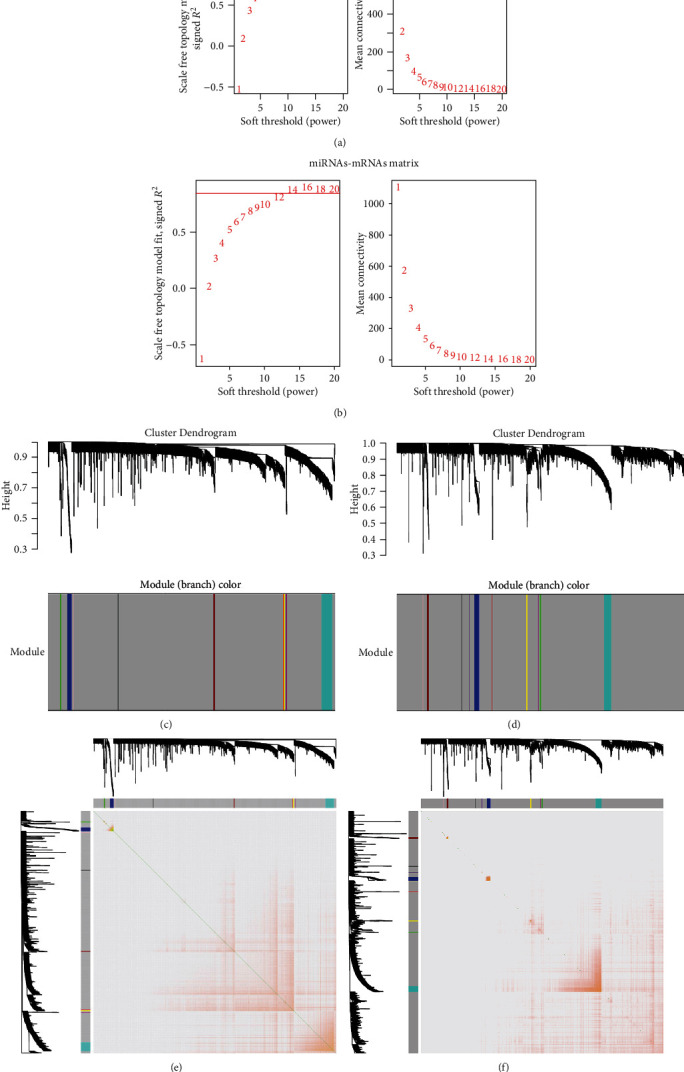
The power of the soft threshold of the (a) lncRNA-miRNA matrix and the (b) miRNA-mRNA matrix; module classification of the (c) lncRNA-miRNA matrix and the (d) miRNA-mRNA matrix; the topological overlap matrix heatmaps of the (e) lncRNA-miRNA matrix and the (f) miRNA-mRNA matrix.

**Figure 5:**
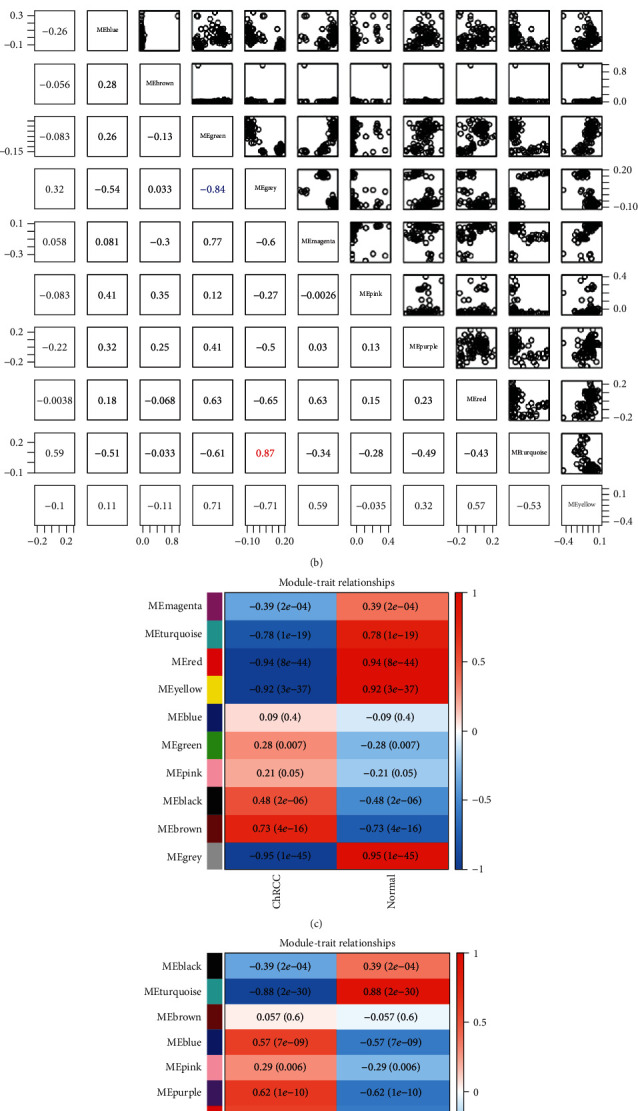
Relationship between module eigengenes of the (a) lncRNA-miRNA matrix and the (b) miRNA-mRNA matrix; module-trait relationships of the (c) lncRNA-miRNA matrix and the (d) miRNA-mRNA matrix; (e) shared mRNAs when predicted.

In the WGCNA, the power of the soft threshold of the lncRNA-miRNA matrix was 10 (Figure 4(a)), and the miRNA-mRNA matrix was 14 (Figure 4(b)), both of which achieved the best satisfaction and consistency of the scale-free R-squared value. After calculating their adjacency and connectivity, these lncRNAs-miRNAs were classified into 10 modules (Figure 4(c)), and miRNAs-mRNAs were classified into 11 modules (Figure 4(d)). Their topological overlap matrix heatmaps are presented in Figures 4(e) and 4(f). Red, yellow, brown, and grey modules of lncRNAs-miRNAs were found to have significant correlation (Figure 5(a)), and greater connections were also observed in green, turquoise, and grey modules of the miRNAs-mRNAs (Figure 5(b)). Modules in these two groups included a total of 1534 DElncRNAs, 98 DEmiRNAs, and 2543 DEmRNAs, which were also more closely related to ChRCC than the others (Figures 5(c) and 5(d)).

When predicting the DElncRNAs and DEmiRNAs of the module genes in miRcode, we identified 116 DElncRNAs (43/73, up/down) and 19 DEmiRNAs (9/10, up/down) and their connective pairs. When using the StarBase, miRTarBase, miRDB, and TargetScan datasets, 512 target mRNAs of the 19 DEmiRNAs were included. Forty-three of them finally coincided with selected module DEmRNAs (Figure 5(e)), which corresponded to 113 lncRNAs and 14 miRNAs.

**Figure 6:**
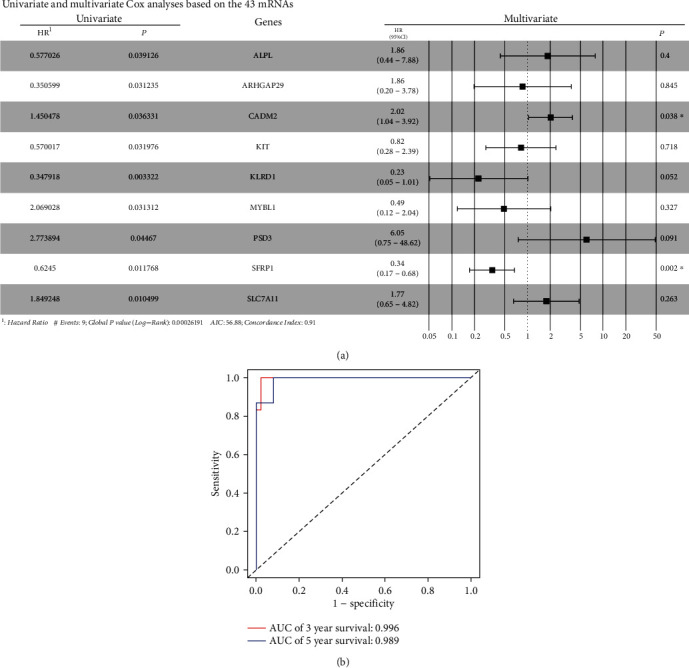
(a) Univariate and multivariate Cox analyses based on the 43 mRNAs; (b) the receiver operating characteristic curve of the model.

**Figure 7:**
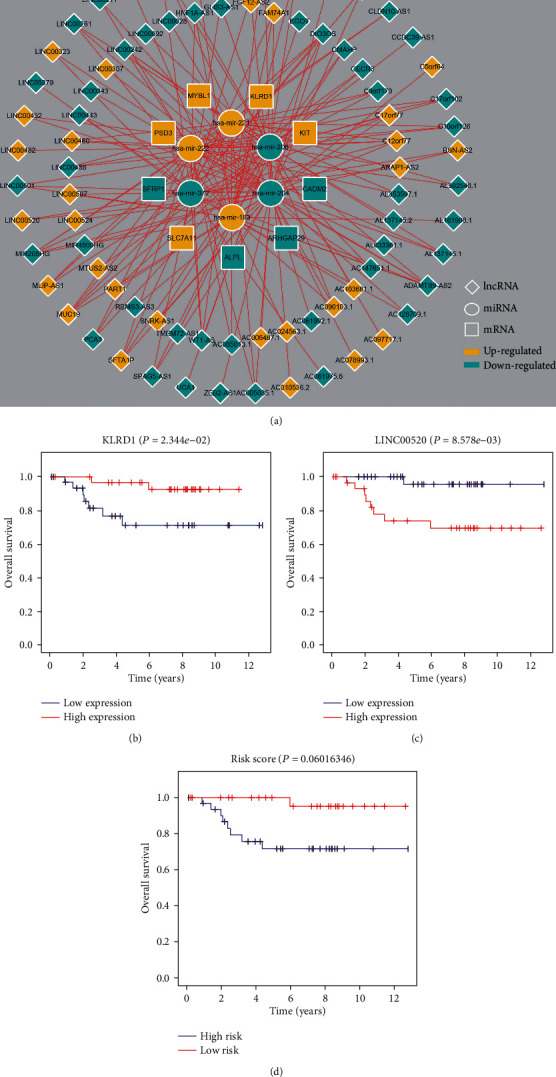
(a) The ceRNA network of ChRCC; Kaplan–Meier curves of (b) KLRD1, (c) LINC00520, and (d) risk score.

Nine mRNAs (ALPL, ARHGAP29, CADM2, KIT, KLRD1, MYBL1, PSD3, SFRP1, and SLC7A11) were identified as prognosis-related genes when a univariate Cox analysis was conducted on the 43 mRNAs (*P* < 0.05). Moreover, the results of multivariate Cox proportional hazards regressions indicted that two of the nine mRNAs (CADM2 and SFRP1) were independent risk factors for ChRCC (Figure 6(a)). The C-index of this model was 0.91, and the 3- and 5-year AUCs (area under receiver operating characteristic curve) were 0.996 and 0.989 (Figure 6(b)), which proved the stability and reliability of the model. Finally, six miRNAs (3/3, up/down) corresponded to 79 lncRNAs (31/48, up/down) and were associated with these nine mRNAs (5/4, up/down). The ceRNA network based on their relationship was constructed using the Cytoscape platform (Figure 7(a)).

Additionally, Kaplan–Meier analyses for the ceRNA members showed that low expression of KLRD1 and high expression of LINC00520 significantly contributed to worse OS for patients with ChRCC (*P* < 0.05) (Figures 7(b) and 7(c)). Meanwhile, the low-risk group also showed obvious superiority over the high-risk group, despite its *P* value being slightly greater than 0.05 (*P* = 0.06016) (Figure 7(d)).

## 4. Discussion

With the progress of molecular biology, the function of noncoding transcriptome has been extensively explored. Multi-RNA competition regulatory networks appear to play indispensable roles in the biological processes and courses of cancer diseases [16, 17]. Several studies have explored and verified ceRNA networks in the past. Wang et al. included 407 normal and 151 acute myeloid leukemia (AML) samples from Genotype-Tissue Expression (GTEx) (https://commonfund.nih.gov/GTEx/) and TCGA datasets in their study. They found that the ceRNA network in AML involved 108 lncRNAs, 10 miRNAs, and 8 mRNAs, which appeared to influence prognosis and cancer progression [18]. Meanwhile, Yao et al. also established a ceRNA network from TCGA database comprising 52 lncRNAs, 17miRNAs, and 79 mRNAs in breast cancer's RNA matrix and in which five lncRNAs were found to significantly affect patients' OS. Furthermore, results of GO and KEGG analyses of these mRNAs were also related to biological characteristics of tumors [19].

WGCNA is a bioinformatic and sensitive method, which is especially suitable for these large and high-dimensional data, as well as for low abundance or fold change genes. It is able to cluster highly related genes from microarray samples into different color modules and explore the relationship between the genes and cancer traits [20]. WGCNA has already been used in various oncological studies to explore hub genes and the regulatory relationships between them [21–23]. In our study, we preformed WGCNA to select highly related module genes, which helped us elucidate the more meaningful RNAs for further prediction.

Importantly, prediction in multiple datasets allowed us to rapidly lockdown the shared high-value genes similar to previous studies [24–27]. Another advantage of our study was the application of univariate and multivariate Cox proportional hazards regressions on selected target mRNAs from which we obtained a reliable and stable prognostic model and identified important genetic biomarkers for ChRCC within the ceRNA network. The excellent C-index and 3- and 5-year survival AUCs further proved the superiority of our model. The Kaplan–Meier curves showed that low-risk patients would achieve better long-term OS.

A member of the cell adhesion molecule gene family, CADM2, has been reported to be underexpressed in the nine mRNAs. This might contribute to the progression of various cancers, including prostate cancer, ovarian cancer, lymphoma, melanoma, and clear renal cell cancer (cRCC) [28–32]. CADM2 is believed to prevent tumor progression, invasion, and metastasis by maintaining cell's polarity and adhesion [32].

Tyrosine protein kinase (KIT) is overexpressed in various cancers [33, 34], especially in ChRCC and oncocytoma. Huo et al. reported that KIT was more sensitive to ChRCC and oncocytoma than other renal cancers, and hence, it would be useful in precise tumor classification and targeted therapy [35, 36]. In the past, SFRP1 has been considered to be a tumor suppressor gene and possibly antagonistic to the wnt signaling pathway [37]. It has been found that increased methylation levels in the SFRP1 promoter region might lead to SFRP1 silencing in cRCC [38, 39]. Meanwhile, low SLC7A11 expression was found to be an important target in the p53 tumor suppression pathway, which is closely related to cell-cycle arrest, apoptosis, and senescence. As the main component of the cystine/glutamate antiporter, underexpressed SLC7A11 could inhibit cellular uptake of cystine and eventually lead to increased cell sensitivity to ferroptosis [40]. Additionally, upregulation of ARHGAP29 might be related to metastasis in gastric cancer [41]. ALPL is primarily related to hypophosphatemia [42]. Rao et al. found that high expression of ALPL led to poor survival outcomes for patients with prostate cancer [43]. However, another study proposed that ALPL could inhibit the motility and aggression of serous ovarian cancer cells [44]. High expression of KLRD1 was reported to inhibit the function of natural killer cells and cytokine-induced killer cells [45, 46]. PSD3 is considered to be a candidate metastasis suppressor gene, and its low expression has been observed to be associated with poor prognosis in ovarian cancer and metastasis in breast cancer [47, 48]. Moreover, MYBL1 is highly expressed in adenoid cystic carcinoma and is often accompanied by genomic rearrangements [49]. These previous findings lend confidence to the hypothesis that the ceRNA network plays an important role in the occurrence and development of cancers. Moreover, to our knowledge, this is the first report regarding the role of these mRNAs in the ChRCC, in which KLRD1 was found by Kaplan–Meier analysis (*P* = 2.344*e* − 2) to significantly affect patients' OS.

Previous studies involving cRCC have reported the importance of the six miRNAs (hsa-mir-222, hsa-mir-204, hsa-mir-206, hsa-mir-183, hsa-mir-372, and hsa-mir-221) in the ceRNA network. In particular, hsa-mir-206, hsa-mir-204, and hsa-mir-372 were found to suppress cancer through corresponding biological functions [50–52], and hsa-mir-183 was considered to be a potential oncogene [53]. Kaplan–Meier analysis also showed that high expression of LINC00520 had an effect on OS. Chen et al., in their study based on the cBioPortal dataset, also emphasized its importance in cRCC [54]. However, more studies are needed to fully explore the biological function of the lncRNAs in ChRCC.

In this study, we constructed a ceRNA network including 79 lncRNAs, 6 miRNAs, and 9 mRNAs. Their possible competitive synergistic biological functions might jointly regulate various processes in ChRCC, and, hence, they may provide new therapeutic targets and a new perspective for ChRCC genetic biology studies. However, there were some limitations to our study. Firstly, the prognostic model of mRNA has not been externally verified. Also, we lacked in vivo and in vitro experiments to verify our results.

## 5. Conclusions

We established the ceRNA network in ChRCC, which included 79 lncRNAs, 6 miRNAs, and 9 mRNAs. Among them, three mRNAs (CADM2, SFRP1, and KLRD1) and one lncRNA (LINC00520) showed promise as potential biomarkers for ChRCC. Our results offer new insights into the diagnosis and treatment of ChRCC and demonstrate the merit of further genetic biology research into ChRCC.

## Figures and Tables

**Table 1:** The clinicopathological characteristics of ChRCC patients.

	Total (*n* = 65)	Alive (*n* = 55)	Dead (*n* = 10)
Gender			
Male	39	32	7
Female	26	23	3
Race			
Asian	2	1	1
White	57	48	9
Black or African American	4	4	0
Not reported	2	2	0
Age at diagnose			
<60 (years)	46	41	5
60-80 (years)	18	13	5
>80 (years)	1	1	0
Mean (SD) (days)	19129.83 (5127.97)	18493.20 (4978.49)	22631.30 (4709.89)
Median (MIN, MAX) (days)	18502 (6556, 31591)	17710 (6556, 31591)	22697 (15045, 28705)
Tumor clinical stage			
Stage I	20	19	1
Stage II	25	23	2
Stage III	14	11	3
Stage IV	6	2	4

**Table 2:** Univariate and multivariate Cox analyses based on the 65 ChRCC patients.

Factors	Univariate analyses				Multivariate analyses			
	HR^1^	Lower.95	Upper.95	*P* value	HR^1^	Lower.95	Upper.95	*P* value
Gender (female reference)								
Male	1.583	0.8987	2.789	0.112	1.5047	0.8142	2.781	0.1923
Race (Black or African American reference)								
Asian	0.7749	0.0847	7.092	0.821	0.4465	0.0405	4.924	0.5103
White	0.5599	0.1988	1.577	0.272	0.4344	0.1406	1.342	0.1474
Not reported	3.6566	0.6303	21.212	0.148	3.6580	0.6043	22.142	0.1580
Age at diagnose (<60-year reference)								
60-80 (years)	0.8579	0.4492	1.639	0.643	0.8596	0.4167	1.773	0.6821
>80 (years)	1.9702	0.2631	14.752	0.509	1.5832	0.1934	12.959	0.6684
Tumor clinical stage (stage I reference)								
Stage II	0.9349	0.5014	1.743	0.8323	0.8648	0.4220	1.772	0.6914
Stage III	1.4956	0.6978	3.206	0.3007	1.3934	0.6012	3.229	0.4392
Stage IV	4.9371	0.9692	25.149	0.0546	6.0728	1.0594	34.811	0.0429^∗^

Signif. codes: 0 “^∗∗∗^” 0.001 “^∗∗^” 0.01 “^∗^” 0.05 “.” 0.1 “ ” 1; ^1^: hazard ratio.

## Data Availability

The dataset supporting the conclusions of this study is available in The Cancer Genome Atlas (TCGA) database.
